# NMDA-receptor inhibition and oxidative stress during hippocampal maturation differentially alter parvalbumin expression and gamma-band activity

**DOI:** 10.1038/s41598-018-27830-2

**Published:** 2018-06-22

**Authors:** Luisa A. Hasam-Henderson, Grace C. Gotti, Michele Mishto, Constantin Klisch, Zoltan Gerevich, Jörg R. P. Geiger, Richard Kovács

**Affiliations:** 1 Institut für Neurophysiologie, Charité – Universitätsmedizin Berlin, corporate member of Freie Universität Berlin, Humboldt-Universität zu Berlin, and Berlin Institute of Health, Berlin, Charité Platz 1, 10117 Berlin, Germany; 2 The NeuroCure Cluster of Excellence, Berlin, Germany; 3 Institut für Biochemie, Charité – Universitätsmedizin Berlin, corporate member of Freie Universität Berlin, Humboldt-Universität zu Berlin, and Berlin Institute of Health, Berlin, Charité Platz 1, 10117 Berlin, Germany; 4 Centre for Inflammation Biology and Cancer Immunology (CIBCI) & Peter Gorer Department of Immunobiology, King’s College London, SE1 1UL London, United Kingdom

## Abstract

Dysfunction of parvalbumin (PV)-expressing interneurons is thought to underlie the alterations of gamma-band oscillations observed in schizophrenia. Although the pathomechanisms of this disease remain unclear, oxidative stress induced by NMDA receptor (NMDAR) hypofunction and decreased glutathione (GSH) synthesizing capacity have been shown to lead to PV-loss and aberrant oscillatory activity. However, the individual contributions of NMDAR-inhibition and GSH-depletion to the developmental alterations observed in schizophrenia are largely unknown. We therefore investigated each condition in isolation using hippocampal slice cultures wherein interneuron maturation occurs entirely *in vitro*. Although both treatments caused oxidative stress, NMDAR-inhibition led to an immediate reduction in gamma oscillation frequency and a delayed loss of PV. In contrast, GSH-depletion immediately decreased PV expression and increased power, without affecting frequency. Hence, although disturbances of PV-expression and gamma oscillations coexist in schizophrenia, they can arise from separate pathological processes.

## Introduction

Schizophrenia is a common neurodevelopmental disorder affecting ~1% of the population with symptoms typically appearing in late adolescence and early adulthood^1^. Current pharmacotherapy non-selectively targets the dopamine receptor D2, primarily ameliorating the positive symptoms (delusions and hallucinations) while improvement of the negative and cognitive symptoms (affective flattening, social withdrawal, memory problems) remains a challenge^1,2^. Information processing and exchange within and between brain regions rely on the synchrony of neural activity, manifested as oscillations in the EEG. In schizophrenia, altered gamma-band activity (30–80 Hz) is indicative of network function disturbances and correlates with the severity of symptoms^3,4^. Parvalbumin positive (PV+) fast-spiking GABAergic interneurons mediate perisomatic inhibition and determine the timing of pyramidal cell firing^5^, which is critical for synchronization of neuronal ensembles during certain forms of gamma activity^6–12^. Altered functional properties of these interneurons, as well as decreased expression of PV and the GABA synthesizing enzyme glutamic acid decarboxylase-67 (GAD67), have been described in individuals affected by schizophrenia and in animal models of the disease^13,14^.

Although the etiology of these alterations is not fully understood, several lines of evidence suggest that NMDAR hypofunction during development might underlie the disturbances of network activity. Numerous genetic risk factors of schizophrenia are related to NMDAR signaling and it has been shown that early postnatal deletion of NMDAR not only disturbs cortical oscillations in the theta/gamma range but also decreases PV and GAD67 expression^15,16^. Previous studies have reported that perinatal NMDAR hypofunction alters interneuron development by eliciting oxidative stress^1^. A direct link between NMDAR hypofunction and oxidative stress was established in a model of acute psychosis induced by the NMDAR antagonist, ketamine, where the loss of the fast spiking phenotype and PV expression in interneurons was associated with the activation of the superoxide producing enzyme, NADPH-oxidase 2 (NOX2)^17^. In addition, the antioxidant N-acetyl-cysteine (Nac) was able to prevent the consequences of NMDAR hypofunction following ventral hippocampus lesion on PV interneuron activity and ameliorated behavioral deficits^18^.

The contribution of oxidative stress to schizophrenia is further supported by the fact that decreased levels of the small molecular antioxidant glutathione (GSH) is a common finding in schizophrenia patients^13,19^, whereas adjunctive treatment with the antioxidant GSH precursor (Nac) improves the negative symptoms^20^. Indeed, polymorphism analysis of the GSH-synthesizing enzyme, glutamate-cysteine ligase, revealed that certain alleles are associated with a higher risk of developing schizophrenia^21^. In animal models, mutations of the GSH-synthesizing enzyme are sufficient to decrease PV expression and change the GABAergic phenotype of interneurons, resulting in aberrant gamma oscillations as well as altered affective behaviour^22,23^.

The positive correlation between synaptic NMDAR activation and GSH synthesis^24^ intuitively links NMDAR hypofunction to decreased GSH levels^25^. Currently, it is still unclear whether the network and interneuron alterations in schizophrenia represent direct consequences of NMDAR hypofunction or if they are downstream from the NMDAR hypofunction-induced oxidative stress.

In this study, we test the hypothesis that NMDAR hypofunction and redox imbalance of the GSH system stereotypically alter the maturation of the neuronal network activity.

Since the alterations in PV+ interneuron properties that underlie the aberrant gamma band activity occur during early perinatal development, we used slice cultures where the maturation of interneurons takes place entirely *ex vivo*^26–29^. Cultures were obtained from transgenic Wistar rats expressing Venus-YFP on the vesicular γ-aminobutyric acid transporter (VGAT) promoter^30^, thereby allowing the identification of changes in the interneuron population following either NMDAR-inhibition or GSH depletion. By assessing total tissue GSH and oxidized protein levels, as well as gamma oscillation properties and PV expression at different time points during development, we investigate whether NMDAR inhibition would induce oxidative stress and alter GSH levels and if the resulting changes in gamma oscillation and PV expression would be comparable to those elicited by direct inhibition of GSH synthesis.

## Results

### Characterization of the *ex vivo* maturation of the hippocampal network activity and PV+ interneurons

In order to investigate the effects of NMDAR inhibition and oxidative stress in isolation, we first characterized the *in vitro* maturation of interneurons and the neuronal network in hippocampal slice cultures.

**Figure 1:**
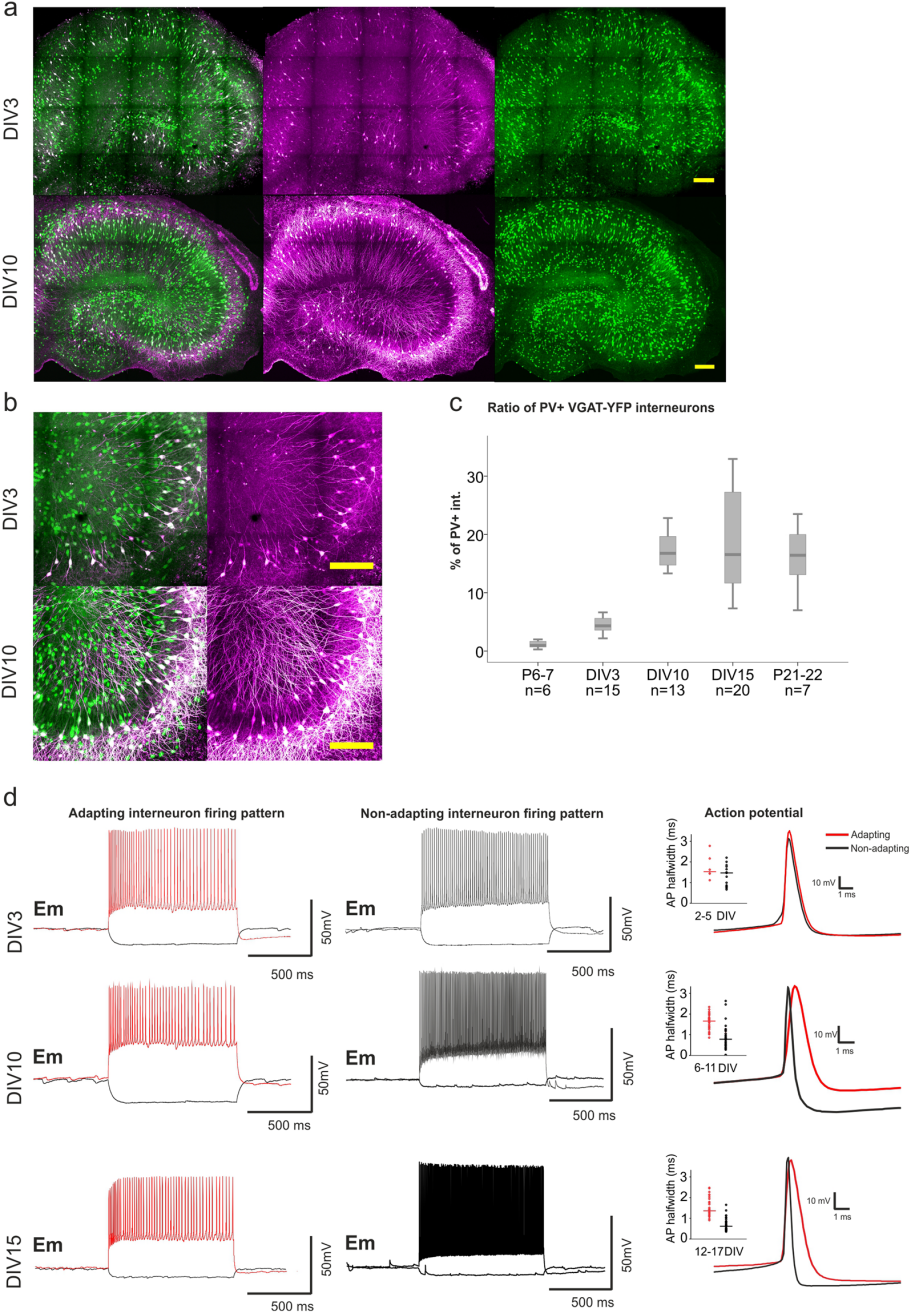
Changes in PV expression and electrophysiological properties of adapting and non-adapting interneurons during slice culture development. Slice cultures of VGAT-YFP rats were labelled for PV at different developmental time points. (**a**,**b**) Representative hippocampal images of DIV3 and DIV10 hippocampal slice cultures (**a**) and respective close ups to the CA3 region are shown (**b**). VGAT-YFP expressing interneurons are displayed in green and PV+ interneurons in magenta (scale bar 200 µm). (**c**) Boxplot depicting in percentage the ratio of YFP+/PV expressing interneurons after 3, 10 and 15 DIV (4.31, 16.76 and 16.54%), and in acute slices at postnatal day (P) 7 and 21–22 (corresponding to DIV15) (0.95 and 16.43%). For each *in vitro* developmental time point hippocampal slices were obtained from 5–6 rats, while for acute slices 3–4 animals were used. The ratio was calculated by counting the total hippocampal number of YFP+ interneurons and PV-labelled interneurons per slice in the 3D reconstruction. (**d**) Exemplary traces of firing patterns upon a 1 s long depolarization step of adapting (red) and non-adapting (black) interneurons are shown for slice cultures at DIV3, 10 and 15. Representative single action potentials of adapting and non-adapting interneurons taken at different developmental stages (DIV2–5; 6–11, 12–17) (right), the excerpt shows the distribution of action potential width at half maximum of AP amplitude (half-width) for respective cell groups. Cells (164) were recorded from 70 slices obtained from 32 animals (for a detailed description of the *in vitro* maturation of electrophysiological properties see Supplementary Fig. 1).

To rule out that the culturing *per se* would alter PV expression in the hippocampus, first we characterized its expression pattern at three different time points i.e. days *in vitro* (DIV) 3 (n = 15), 10 (n = 13) and 15 (n = 20) and compared it to that of age matched acute slices (at P7 (n = 6) and P21–22 (n = 7)), which would represent the intact development of PV expression). Even though PV+ interneuron migration is complete by the time of slice preparation (P7), PV identification is limited as the protein is still barely expressed^31^. Using multiphoton microscopy and 3D reconstructions of the entire hippocampal slice culture, we quantified for each slice the total hippocampal number of YFP+ and PV-labelled interneurons and calculated the ratio of YPF+ interneurons expressing PV (presented in percentage). Indeed, we observed a time-dependent increase of PV labelling following explantation at P7 (DIV0), time point at which only 1% of the interneurons were PV+. By DIV3, 4% of the interneurons expressed PV, rising to 17% by DIV10 (Fig. 1a–c). This percentage remained stable at DIV15 (16.5%). These values were comparable to those observed in age-matched acute slices (16.4%) (P21–22 vs DIV15; p = 0.699; Fig. 1c), indicating that, despite the model-intrinsic slice thinning and broadening of the pyramidal cell layers during cultivation, PV expression continues to develop in slice cultures.

At the age of slice explantation, the electrophysiological properties of PV+ interneurons decisively differ from the adult characteristics, which in rodents reach complete maturity around postnatal day 25–28^4,32^. Since in our experimental setting the maturation process takes place under culture conditions, we first characterized the developmental changes in passive and active electrophysiological properties of VGAT-YFP-interneurons. Whole-cell patch clamp recordings were carried out at different time points ranging from DIV2 to 17. Interneurons recorded from the str. pyramidale - str. oriens border were divided into adapting and fast-spiking/non-adapting interneurons, based on their firing pattern (inter-event intervals between the first and last five action potentials (AP) in an AP train) (Fig. 1d). With increasing DIV, both interneuron groups showed a statistically significant (p < 0.01) negative correlation of membrane resistance (two-tailed Spearman correlations, ρ: −0.485 and ρ: −0.351) and a positive correlation of membrane capacitance (ρ: 0.368 and ρ: 0.306, for non-adapting and adapting, respectively) likely indicating neuronal growth/arborization in culture (Supplementary Ia and b). In adapting interneurons, AP-frequency and AP-half-width did not change significantly. By contrast, in non-adapting interneurons the maximum AP-frequency and AP-half-width showed a strong correlation with DIV, where the maximum frequency became higher (ρ: 0.657) and the APs narrower (ρ: −0.428), reaching a value of 152.5 ± 8 Hz and 0.7 ± 0.03 ms after 9 days in culture (p < 0.01). These parameters remained stable up to DIV15 (Supplementary Ic and d).

**Figure 2:**
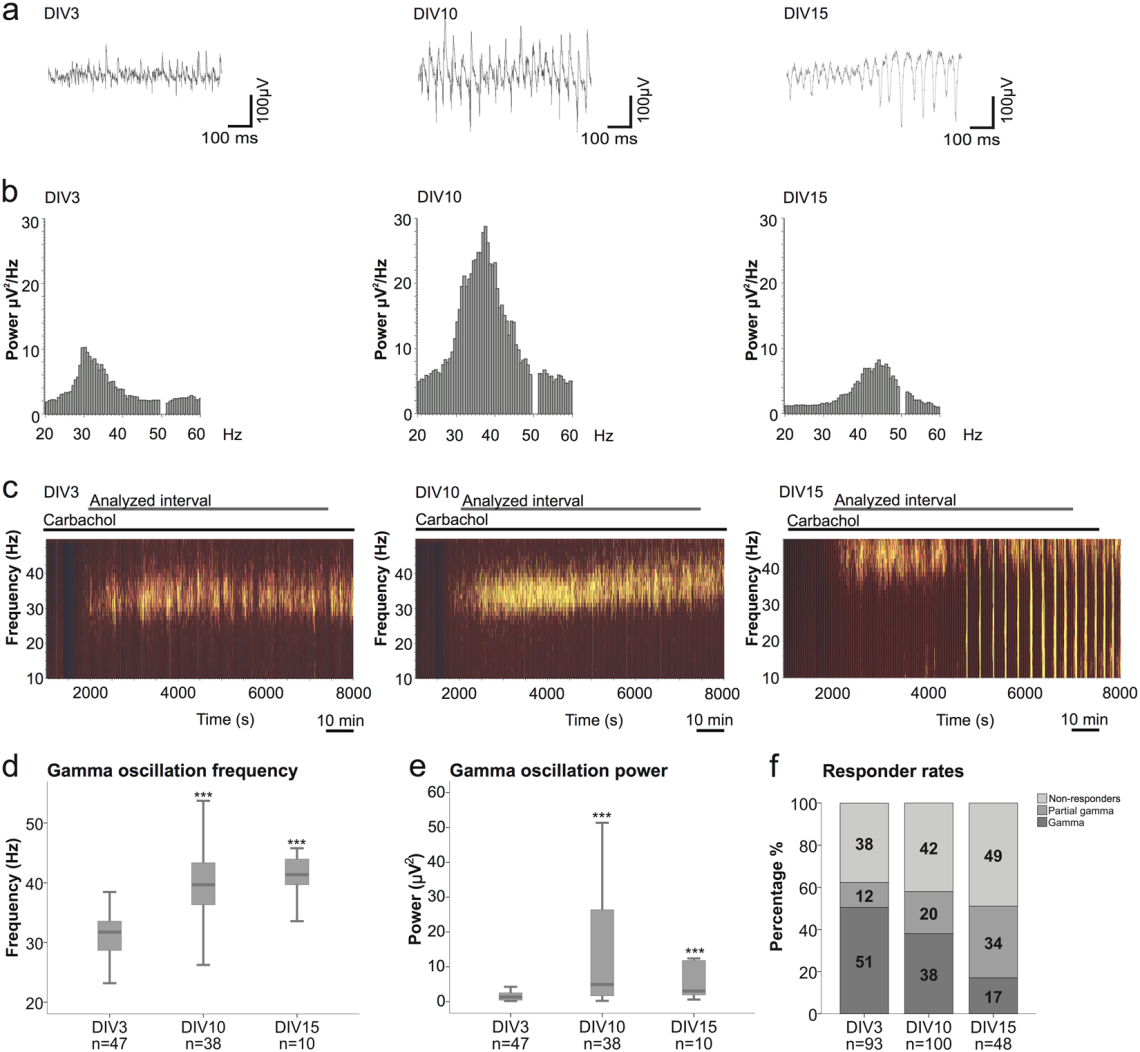
*In vitro* maturation of network activity. (**a**) Exemplary field potential traces of carbachol (Cch) induced gamma oscillations at DIV3 (left), DIV10 (middle) and DIV15 (right). In the DIV15 excerpt, the transition from gamma oscillations to epileptiform discharges are depicted. (**b**) Representative power spectra (window size: 120 s) of gamma oscillations at DIV3 (left), DIV10 (middle) and DIV15 (right). (**c**) Representative sonograms of local field potential recordings obtained from DIV3 (left), DIV10 (middle) and DIV15 (right) slices during gamma oscillations. An example of gamma oscillations intermingled with spontaneous discharges observed in DIV15 cultures is shown in the right sonogram. (**d**,**e**) Boxplots presenting the gamma oscillation median of peak frequency and power in a 90 min period (from 2000–7400 s recording interval) at DIV3, 10 and 15 (DIV3: 31.7 Hz, 1.3 µV^2^, DIV10: 39.7 Hz, 4.9 µV^2^, DIV15: 41.4 Hz, 3 µV^2^). The number of slices (n) are indicated in the plots. The number of animals used are as follows: 30, 31 and 8 rats for DIV3, 10 and 15, respectively. Kruskal-Wallis test and post-hoc Mann-Whitney U test was used for the comparison between DIV3 and DIV10/15 (***p < 0.001). (**f**) Responder rates of Cch-induced gamma oscillations are described for the three developmental time points (DIV3, 10 and 15). The number of slices displaying continuous gamma oscillations (gamma), discontinuous gamma oscillations (partial gamma) and non-responder slices (non-responders) are presented in percentage. For statistical analysis the two-tailed Fisher’s exact test was performed.

We assessed the development of the hippocampal network by performing local field potential recordings (CA3) of carbachol (Cch)-induced gamma oscillations at three developmental time points: DIV3, 10 and 15. The peak frequency and power of a recording interval of 90 minutes (from 2000–7400 s) were calculated for the slices displaying oscillatory activity. We observed an increase in both, peak frequency and power from DIV3 to DIV10, which remained stable by DIV15 (Fig. 2d and e). The peak frequency rose from a median of 32 Hz at DIV3 to 40 Hz at DIV10 (p = 5.62^−10^, n = 47 and 38, Mann-Whitney U test) with no further changes by DIV15 (41 Hz, n = 10, p = 0.4536, Mann-Whitney U test). Similarly, the peak power increased significantly from DIV3 to DIV10 (Mdn 1.3 µV^2^, 4.9 µV^2^, p = 1.5^−5^, n = 47 and 38, Mann-Whitney U test), while no significant increase was observed between DIV10 and 15 (Mdn 4.9 µV^2^, 3.1 µV^2^, n = 38 and 10, p = 0.4022, Mann-Whitney U test) (Fig. 2d and e) (Detailed statistical analysis is shown in supplementary material). Interestingly, the ability of the cultures to sustain continuous gamma oscillations throughout the 90 min period declined gradually with time in culture. At DIV3, 51% of the slices showed persistent oscillations dropping to only 17% at DIV15 (Fig. 2f). Longer time in culture induced a shift to shorter periods of gamma oscillations (partial gammas), which were often followed by a switch into recurrent discharges, resembling epileptiform activity (Fig. 2a right and c right). Only slices with continuous gamma oscillations for the given 90 min interval were included in the analysis. Thus, extending the study until DIV15 did not provide additional information, due to increasing propensity to epileptiform discharges.

Fast-spiking PV+ interneurons are indispensable for maintaining synchronicity during Cch-induced gamma oscillations in the CA3 region^6–9^. Therefore, the gradual increase in frequency between DIV3 and DIV10 might correlate with the observed changes in the electrophysiological properties of non-adapting interneurons. Together, these results suggested that interneuron and network maturation reaches a plateau by DIV10, suggesting an experimental window for treatment prior to this time point.

### Drug application for NMDAR inhibition and induction of oxidative stress

Following the characterization of the maturation of hippocampal slice cultures, we proceeded to simulate NMDAR hypofunction or GSH-depletion in a developmental window from DIV1-10. Slice cultures were exposed either to the selective NMDAR antagonist DL-2-Amino-5-phosphonopentanoic acid (APV, 50 µM), or to a combined application of buthionine sulfoximine (BSO), an inhibitor of γ-glutamylcysteine synthetase (γ-GCS), the rate-limiting enzyme of GSH synthesis, and Auranofin (Au), a thioredoxin reductase inhibitor^33^. We have chosen to block the Peroxiredoxin/Thioredoxin/Thioredoxin-reductase antioxidant system (TRX) in parallel to γ-GCS, as it was shown to contribute to the peroxide detoxification^33^, and to compensate for the effect of GSH deprivation^34,35^. The short and long term effects of each treatment on total tissue GSH content, oxidized protein levels, gamma oscillations and PV expression were assessed at an early (DIV3) and a late (DIV10) developmental stage. For all cases, slices were exposed to the inhibitors during the entire culture period starting from DIV1 but they were withdrawn during the induction and recording of gamma oscillations.

### NMDAR inhibition increases protein oxidation without affecting GSH levels

**Figure 3:**
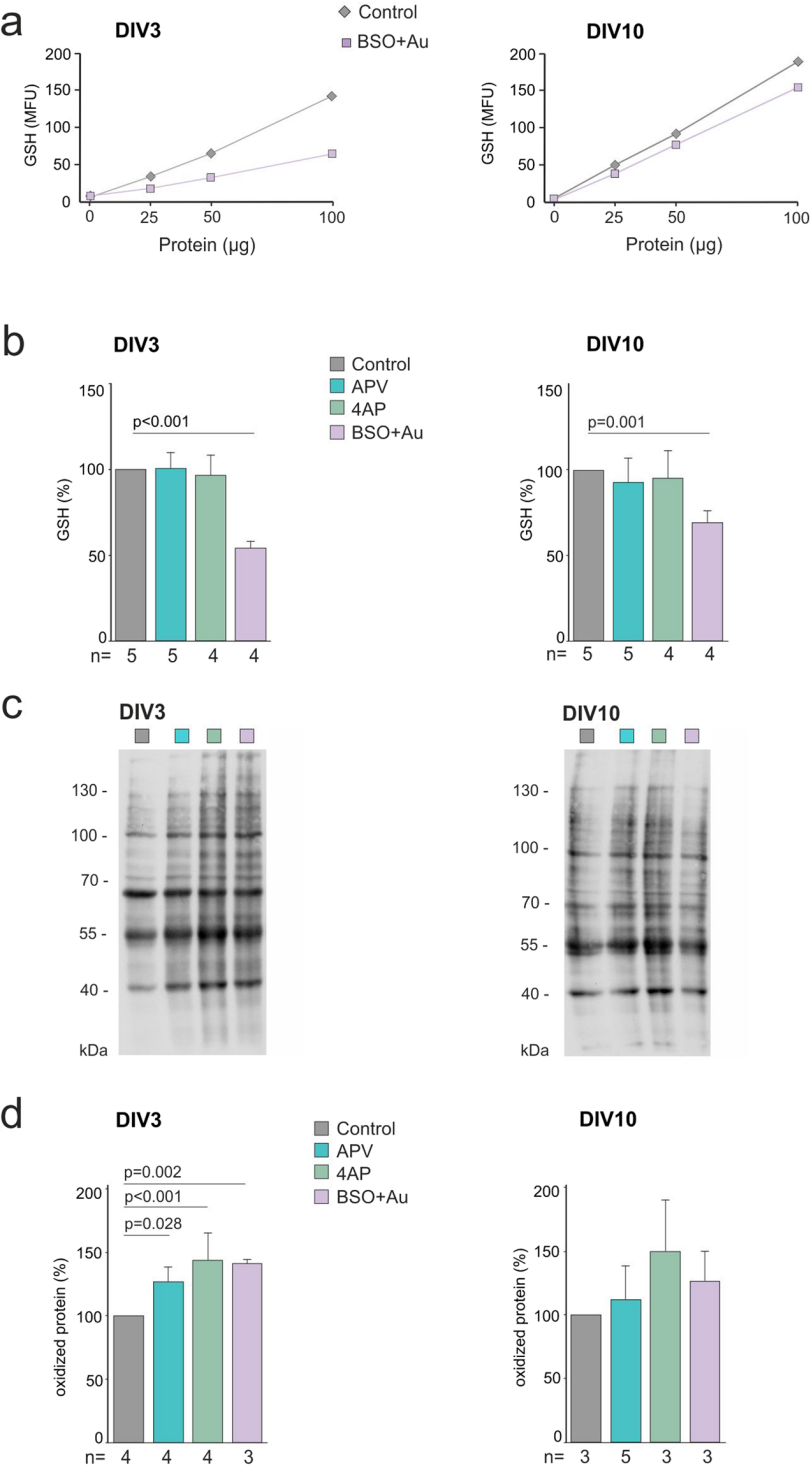
Effects of NMDAR inhibition, oxidative stress and increased synaptic activity on total GSH content and on protein oxidation levels. (**a**) Representative examples of the linear correlation between the GSH levels (measured as mBCI fluorescence) and the protein concentration in the cell lysate of DIV3 (left) and 10 (right) in control and BSO/Au treated (oxidative stress) slice cultures. (**b**) Quantification of the relative GSH content after APV, 4AP and BSO/Au treatments at DIV3 (left) (Control 100; APV 102.8 ± 8.7; 4AP 96.5 ± 12; BSO/Au 55.3 ± 4.3, p = 8.12^−8^, mean ± SD, ANOVA, post-hoc Bonferroni) and DIV10 (right) (Control 100; APV 93 ± 14.4; 4AP 95.4 ± 16.2; BSO/Au 72.3 ± 8.9, p = 0.001, mean ± SD, ANOVA, post-hoc Bonferroni). All treatments started at DIV1 and the drugs were present throughout the development. The number of independent experiments (n) are shown in the figures, for each experiment (4 or 5) 9–18 slice cultures from 3–4 rats were used. (**c**) Representative oxyblot (of 3–4 independent experiments) presenting oxidized protein level in cell lysates at DIV3 (left) and 10 (right) in control and treated slice cultures. Full-length gels are included in the supplementary material. (**d**) Relative oxidized protein levels in control and treated slice cultures at DIV3 (left) (Control 100; APV 126.8 ± 11.5, p = 0.028; 4AP 144.1 ± 19.1, p = 3.5^−5^; BSO/Au 141.2 ± 4, p = 0.002; mean ± SD, ANOVA, post-hoc Bonferroni) or DIV10 (right) (Control 100; APV 112.1 ± 26.3; 4AP 149.7 ± 40.2; BSO/Au 126.6 ± 23.2, ANOVA, post-hoc Bonferroni). For each experiment (n) 9–18 slice cultures from 3–4 rats were used. Significant differences between groups are marked with the respective p values (Anova test with Bonferroni correction for multiple comparisons).

In the first set of experiments, we investigated whether NMDAR inhibition would induce oxidative stress and reduce the total tissue GSH content, comparable to the partial blockade of the GSH synthesis by BSO. In order to determine a tolerable level of GSH-depletion that would not interfere with slice viability, four different BSO concentrations (0.5, 1, 5, 10 µM) were tested. While BSO was lethal at concentrations above 5 µM, inducing swelling and cell loss after overnight exposure, slices treated with BSO/Au (1 µM–1 µM) remained viable for as long as DIV15. GSH levels were determined spectrophotometrically from slice lysates by measuring the fluorescent adduct of monochlorobimane (mBCI) following the reaction with glutathione-S-transferase^24^. BSO/Au exposure resulted in a ~45% reduction of GSH levels at DIV3 (GSH level was 55.3 ± 4.3% of the control, n = 5, 4 independent experiments for control and BSO/Au, respectively, p < 0.001, mean ± SD, ANOVA, Bonferroni post-hoc) and 31% at DIV10 (GSH level was 72,3 ± 8.9% of the control, n = 5, 4, independent experiments for control and BSO/Au, p = 0.001, ANOVA, Bonferroni post-hoc) (Fig. 3b). In contrast, NMDAR inhibition by APV did not change the total tissue GSH levels, neither at DIV3 (n = 5) nor at DIV10 (n = 5), suggesting that NMDAR activity is not indispensable for the maintenance of basal GSH synthesis (Fig. 3b). Subsequent confocal imaging of the fluorescent mBCI-GSH adduct in intact slice cultures confirmed previous studies reporting higher cytosolic GSH concentrations in putative glial cells than in neurons^36^ (Supplementary Fig. 2a). Thus, the inability of APV to change GSH levels indicates that total tissue GSH is largely determined by the glial compartment and independent of NMDAR activity.

Despite the unchanged GSH levels, NMDAR inhibition might still induce oxidative stress in neurons, which might have remained undetected in the previous experimental setting. We therefore proceeded to assess protein oxidation in slice cultures via oxyblot assay, which labels the carbonyl groups introduced into proteins by oxidative reactions. We observed an increase in protein oxidation at DIV3 in both the APV and BSO/Au treated groups (APV: 126.8 ± 11.5%, p = 0.028; BSO/Au, 141.2 ± 4%, p = 0.002, mean ± SD, oxidized proteins in percentage of the control, n = 4, 4, 3 independent experiments, ANOVA, Bonferroni post-hoc), as compared to age-matched control slice cultures (Fig. 3d). However, despite the continuous exposure to the inhibitors, the effects were not maintained and no significant increase in oxidized proteins was observed at DIV10 (APV: 112.1 ± 26.3%, p = 1; BSO/Au: 126.6 ± 23.2%, mean ± SD, p = 1, n = 3, 5, 3 independent experiments, ANOVA, Bonferroni post-hoc) (Fig. 3d). To investigate whether the initial increase might be intrinsic to the slice model and the culturing conditions, we analyzed the time course of protein oxidation (DIV0 4, 7, 10) in untreated cultures. The amount of oxidized proteins gradually increased following explantation (P7-DIV0), peaking at DIV7 and recovering to a less oxidized state at DIV10 (Supplementary Fig. 2b). These results point to the presence of an intrinsic adaptive antioxidant response and enhanced removal of oxidized proteins after DIV7, probably also boosting the recovery from the oxidative stress induced by BSO/Au or APV.

### NMDAR antagonism decreases the frequency of gamma oscillations before reducing PV expression

**Figure 4:**
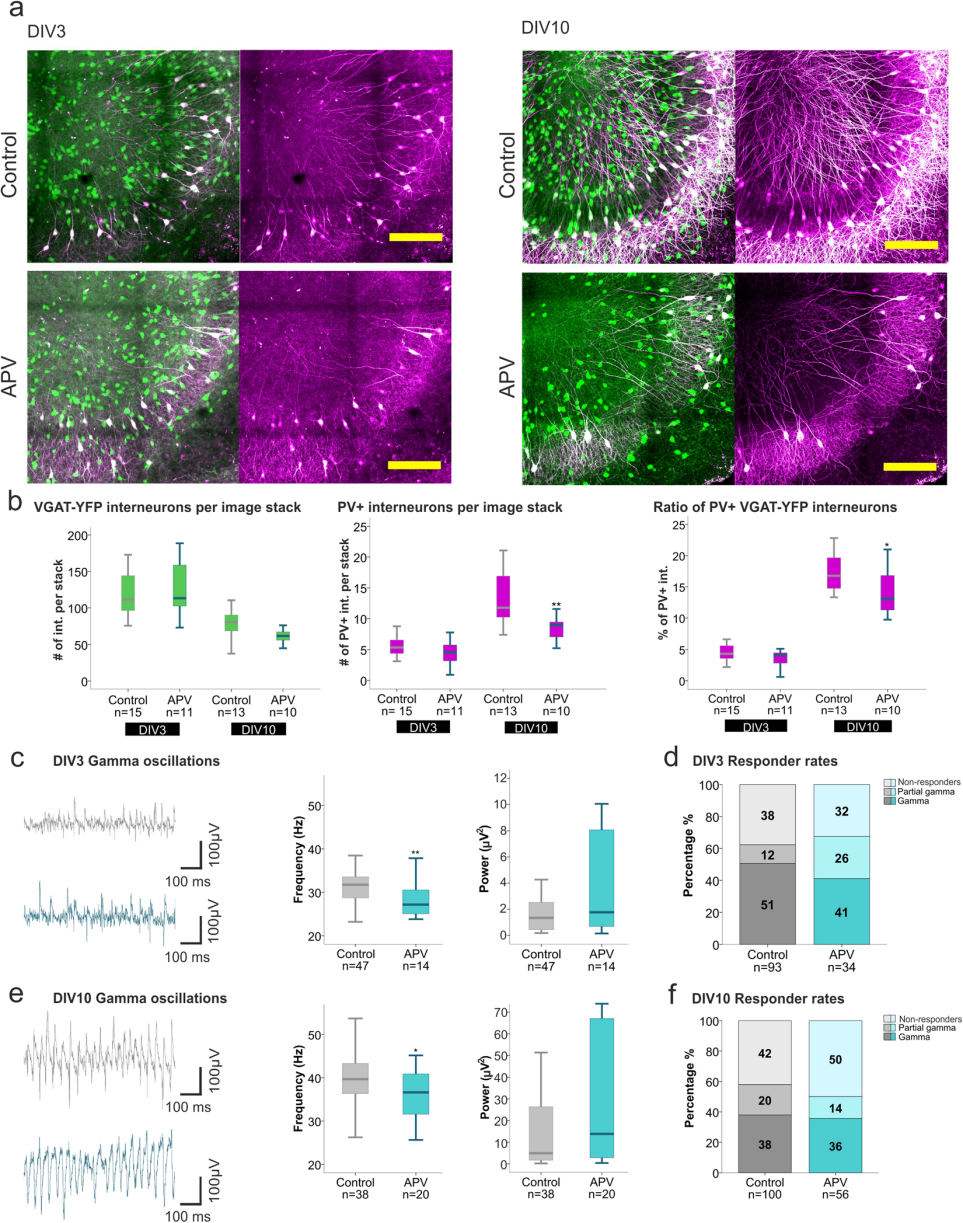
Effects of NMDAR inhibition on PV expression and gamma oscillations. (**a**) Representative images of the hippocampal CA3 region of control and APV treated slices at DIV3 (left) and 10 (right), depicting VGAT-YFP interneurons (green) and PV labelled interneurons (magenta) (scale bar 200 µm). (**b**) Boxplots presenting the median of the number of VGAT-YFP interneurons (left) (DIV3 control 111.8, APV 113.2, DIV10 control 80.5, APV 61.8) and PV+ interneurons (middle) (DIV3 control 5.3, APV 4.5, DIV10 control 11.8, APV 8.9) per focal plane as well as the ratio of PV+/YFP+ interneurons in the 3D hippocampal slice (right) (DIV3 control 4.3%, APV 4.1%, DIV10 control 16.8%, APV 13.1%) for control and APV treated slices, at DIV3 and 10. Hippocampal slices were prepared from 5–6 rats per group. The Mann-Whitney U test was used for statistical analysis (*p < 0.05, **p < 0.01). (**c**,**e**) Boxplots presenting the gamma oscillation peak frequency and power of control and APV treated slices at DIV3 (**c**) (control 31.7 Hz, 1.3 µV^2^, APV 27.2 Hz, 1.8 µV^2^) and DIV10 (**e**) (control: 39.7 Hz, 4.9 µV^2^; APV: 36.6 Hz, 13.8 µV^2^). The number of animals used for the control and APV-treated slice preparation are as follows: DIV3; 30 and 10 rats, DIV10; 31 and 13 rats. Statistical analysis was performed using the Mann-Whitney U test on the respective median values (calculated from the 2000–7400 s interval) (*p < 0.05, **p < 0.01). (**d**,**f**) Responder rates to Cch of control and APV treated slice cultures at DIV3 (**d**) and 10 (**f**). Statistical analysis was performed using the two-tailed Fisher’s exact test including the three categories of activity (gamma, partial gamma and non-responders).

Previous studies have reported a decrease in PV expression after acute and chronic NMDAR hypoactivity *in vivo* in different brain regions^15,37^. Therefore, we investigated whether the same trend could be observed in our model. Exposing slices to APV gradually decreased PV expression reaching statistical significance by DIV10 (Control Mdn 16.8, n = 13; APV Mdn 13.1, n = 10, p = 0.0256, Mann-Whitney U test) (Fig. 4a and b right). At this developmental time point a tendency to a decrease in the absolute number of interneurons was detectable when compared to controls (Control Mdn 80.5; APV Mdn 61.8, p = 0.0628, Mann-Whitney U test), indicating a long-term negative effect of NMDAR inhibition on the interneuron survival (Fig. 4b left).

Previous evidence suggests that the loss of PV goes along with alterations in the electrophysiological properties of interneurons^15,17^. In the next set of experiments, we investigated whether the decreased PV expression induced by APV would also reflect on the hippocampal network activity. Already by DIV3, we observed a marked decrease in the peak frequency of the Cch-induced gamma oscillations (Control Mdn 31.7 Hz, n = 47; APV Mdn 27.2 Hz, n = 14, p = 0.0031, Mann-Whitney U test), despite the absence of a significant change in PV expression or absolute numbers of interneurons. This decrease in frequency was maintained up to DIV10 (Control Mdn 39.7 Hz, n = 38; APV Mdn 36.6 Hz, n = 14, p = 0.0254, Mann-Whitney U test). No overt changes in the power of gamma oscillations were detected at any assessed time point (Fig. 4c and e). Detailed statistics are included in supplementary material. Furthermore, NMDAR inhibition did not alter the propensity of the slices to develop gamma oscillations, which was comparable to that of the control groups (DIV3 p = 0.1543, n = 93 and 34; DIV10 p = 0.5733, n = 100 and 56, Fisher’s exact test two-tailed) (Fig. 4d and f).

Thus, NMDAR inhibition in slice cultures emulates the reduced PV expression and altered gamma oscillation frequency observed *in vivo* after perinatal exposure to NMDAR antagonists^38^. However, in our setting, the alterations in the gamma-band activity precede the loss of PV expression in interneurons.

### Oxidative stress disturbs PV expression while transiently increasing gamma oscillation power

**Figure 5:**
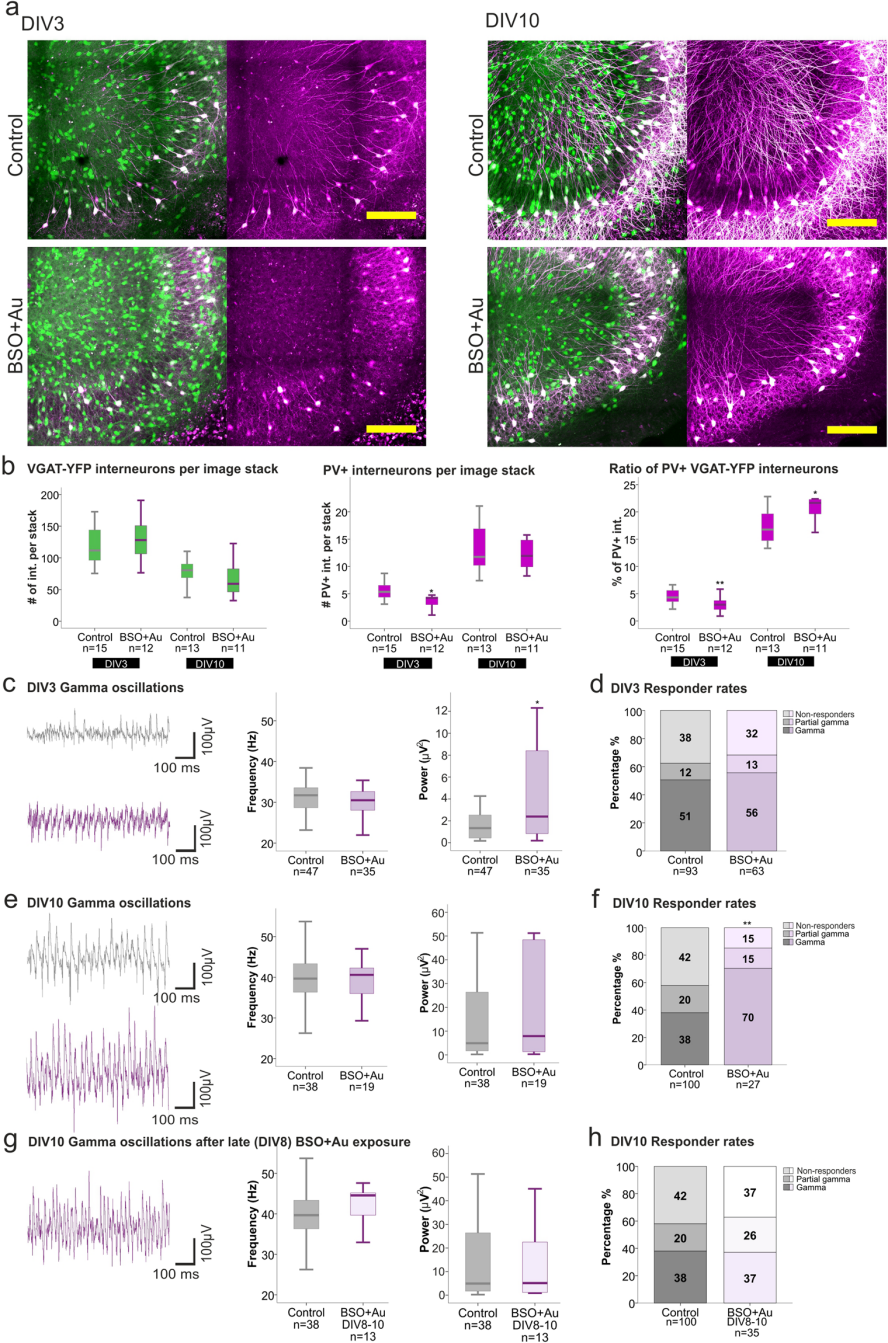
Effects of oxidative stress on PV expression and gamma oscillations. (**a**) Representative images of the hippocampal CA3 region of control and BSO/Au treated slices at DIV3 (left) and 10 (right), depicting VGAT-YFP interneurons (green) and PV+ interneurons (magenta) (scale bar 200 µm). (**b**) Boxplots presenting the median of the number of VGAT-YFP interneurons (left) (DIV3 control 111.8, BSO/Au 128.1, DIV10 control 80.5, BSO/Au 59.1) and PV+ interneurons (middle) (DIV10 control 5.3, BSO/Au 4.2, DIV10 control 11.8, BSO/Au 11.9) per focal plane as well as the ratio of PV+/YFP+ interneurons in the 3D hippocampal slice (right) (DIV3 control 4.3%, BSO/Au 3%, DIV10 control 16.8%, BSO/Au 21.7%) for control and BSO/Au treated slices, at DIV3 and 10. Hippocampal slices were prepared from 5–6 rats per group. The Mann-Whitney U test was used for statistical analysis (*p < 0.05, **p < 0.01). (**c**,**e**,**g**) Boxplots present the median of the gamma oscillation peak frequency and power of control and BSO/Au treated slices at DIV3 (**c**) (control: 31.7 Hz, 1.3 µV^2^, BSO/Au: 30.5 Hz, 2.3 µV^2^) and DIV10 (**e**) (control: 39.7 Hz, 4.9 µV^2^; BSO/Au: 40.6 Hz, 7.9 µV^2^). The peak frequency and power of gamma oscillations in slices exposed to BSO/Au in later stages of development (from DIV8-10) are also displayed (control: 39.7 Hz, 4.9 µV^2^; BSO/Au 44.6 Hz, 5.1 µV^2^) (**g**). The number of animals used for the control, early exposure BSO/Au treatment and late exposure BSO/Au treatment are as follow: DIV3; 30 and 9 rats, DIV10; 31, 15 and 6 rats. Statistical analysis was performed using the Mann-Whitney U test on the medians (calculated from the 2000–7400 s recording interval) of responding slices (*p < 0.05). (**d**,**f**,**h**) Responder rates to Cch of control and BSO/Au treated slices recorded at DIV3 (**d**) and 10 (**f**), as well as for late BSO/Au treatment (DIV8-10) (**h**) are given as percentage of all slices investigated. Statistical analysis was performed using the two tailed Fisher’s exact test including the three categories of activity (gamma, partial gamma and non-responders).

In the following set of experiments we investigated whether oxidative stress alone would mimic the alterations observed following NMDAR inhibition. Partial inhibition of the GSH synthesis and Trx-reductase activity reduced the number of PV+ interneurons at DIV3 (Control Mdn 5.3, n = 15; BSO/Au Mdn 4.2, n = 12, p = 0.0112, Mann-Whitney U test) without affecting the overall number of interneurons (Fig. 5a and b left and middle). The remaining PV+ interneurons presented truncated dendrites. However, at DIV10 the PV expression was increased again and the PV+/YFP ratio was restored to control levels. While at this time point the number of PV+ interneurons per focal plane in treated slices was similar to that observed in controls (Control Mdn 11.8, n = 13; BSO/Au Mdn11.9, n = 11, p = 0.7856, Mann-Whitney U test), the rise in the ratio hints to a moderate interneuron loss still not reaching significance (Control Mdn 80.5, n = 13; BSO/Au Mdn 59.1, n = 11, p = 0.2767) (Fig. 5a and b left and middle). This lower interneuron count together with the unchanged number of PV+ interneuron explains the significant increase in the PV+/YFP+ interneurons ratio (Control Mdn 16.8, n = 13; BSO/Au 21.7, n = 11, p = 0.0190, Mann-Whitney U test) (Fig. 5b right). In fact, we observed an apparent thinning of slice cultures after long exposure to oxidative stress, suggesting general cell loss. Despite the marked reduction of PV expression after initial BSO/Au exposure (DIV3), the frequency of gamma oscillations remained unchanged while the power significantly increased (Control Mdn 1.3 µV^2^, n = 47; BSO/Au Mdn 2.39 µV^2^, n = 35, p = 0.0273, Mann-Whitney U test) (Fig. 5c and d), as opposed to the observations in cultures following NMDAR inhibition. Although at DIV10 the peak frequency and power remained similar to control levels, the prolonged oxidative stress almost doubled the responder rates, suggesting a higher ability to maintain continuous gamma band activity for periods longer than 90 min (Control 38%, n = 100 vs BSO/Au 70%, n = 56, p = 0.0094, two-tailed Fisher’s exact test) (Fig. 5f).

As the disturbance in PV expression and the increased gamma power were only present following two-day exposure to BSO/Au, we proceeded to investigate whether these alterations were dependent on the developmental stage of the cultures or if they solely represented a stereotypic acute response to oxidative stress. To this end, we administered BSO/Au to DIV8 slices and assessed alterations of gamma oscillations at DIV10. In these slices the gamma frequency and power did not differ from those observed in the control group (Fig. 5g and h), indicating that the electrophysiological effects of the inhibition of GSH/Trx systems strongly depends on the developmental state of the neuronal network.

Based on these findings, we concluded that NMDAR inhibition and oxidative stress differentially alter network development with respect to the time course of PV reduction and the frequency of the gamma-band oscillatory activity

### Increasing synaptic transmission improved interneuron survival despite enhanced protein oxidation

Activation of synaptic NMDAR is suggested to increase the capability of neurons to synthesize GSH and to cope with oxidative stress^24,39^. In the last set of experiments, we sought to determine if a general increase in neuronal activity would affect tissue GSH levels and reverse the effects of oxidative stress on the network maturation. To this end, we applied 4AP, a broad-spectrum inhibitor of voltage-gated potassium channels. Despite the enhanced network activity, GSH levels remained unchanged at both DIV3 (p = 0.620) and DIV10 (p = 0.339) (Fig. 3b and c). Remarkably, 4AP induced a significant increase in oxidized protein levels at DIV3 (p = 0.019). Nevertheless, at DIV10, the statistical significance was lost (Fig. 3c and d), which can be explained by the model-inherent decline of protein oxidation after the first week in culture (Supplementary Fig. 2). Thus, enhancing network activity with 4AP did not alter the GSH synthesis but rather increased oxidative stress in our paradigm^40–42^.

**Figure 6:**
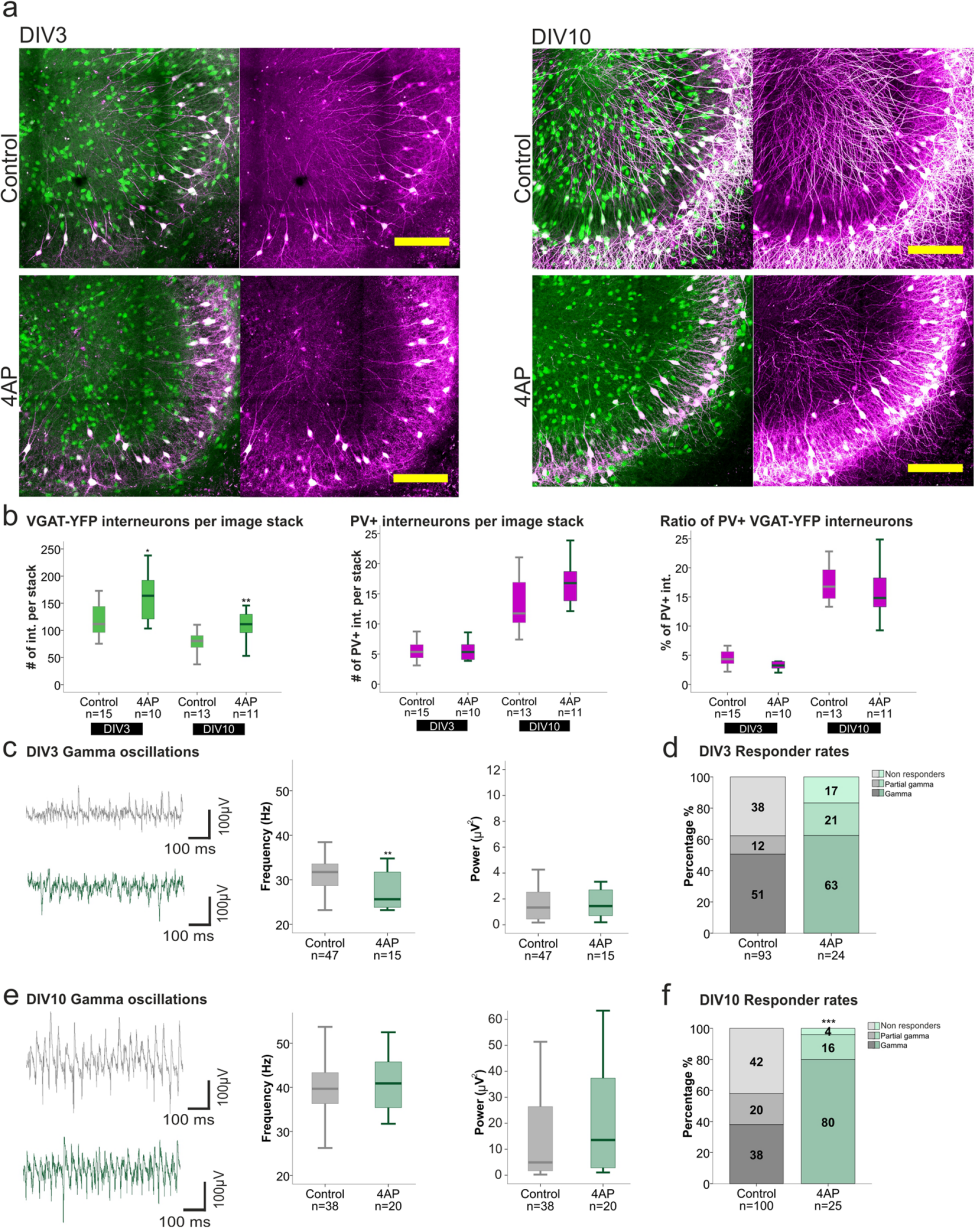
Effects of increased network activity on PV expression and gamma oscillations. (**a**) Representative images of the hippocampal CA3 region of control and 4AP treated slices at DIV3 (left) and 10 (right), depicting VGAT-YFP interneurons (green) and PV+ interneurons (magenta) (scale bar 200 µm). (**b**) Boxplots presenting the median of the number of VGAT-YFP interneurons (left) (DIV3 control 111.8, 4AP 163.8, DIV10 control 80.5, 4AP 111.4) and PV+ interneurons (middle) (DIV3 control 5.4, 4AP 5.3, DIV10 control 11.8, 4AP 16.8) per focal plane as well as the ratio of PV+/YFP+ interneurons in the 3D hippocampal slice (right) (DIV3 control 4.3%, 4AP 3.2%, DIV10 control 16.8%, 4AP 14.8%) for control and 4AP treated slices, at DIV3 and 10. Hippocampal slices were prepared from 5–6 rats per group. The Mann-Whitney U test was used for statistical analysis (*p < 0.05, **p < 0.01). (**c**,**e**) Boxplots present the median of the gamma oscillation peak frequency and power of control and 4AP treated slices at DIV3 (**c**) (control: 31.7 Hz, 1.3 µV^2^, 4AP: 25.6 Hz, 1.5 µV^2^) and DIV10 (**d**) (control: 39.7 Hz, 4.9 µV^2^; 4AP: 40.9 Hz, 13.5 µV^2^). The number of animals used for control and 4AP treated slices are as follow: DIV3; 30 and 10 rats, DIV10; 31 and 10 rats. Statistical analysis was performed using the Mann-Whitney U test on the medians of responding slices (**p < 0.01). (**d**,**f**) Responder rates to Cch of control and 4AP treated slices recorded at DIV3 (**d**) and 10 (**f**) are presented in percentage. Statistical analysis was performed using the two-tailed Fisher’s exact test including the three categories of activity (gamma, partial gamma and non-responders).

As PV expression and the fast-spiking phenotype of PV+ interneurons develop in an activity-dependent manner^28,43,44^, we tested how the increased neuronal activity influenced these parameters in our model. Exposure to 4AP increased the number of interneurons throughout the culturing period (DIV3 control Mdn 111.8, n = 15; 4AP Mdn 163.8, n = 10, p = 0.0229; DIV10 control Mdn 80.5, n = 13; 4AP Mdn 111.4, n = 11, p = 0.0050, Mann-Whitney U test) (Fig. 6a and b), whereas the ratio of PV+/YFP+ interneurons did not change. As with NMDAR inhibition, 4AP also reduced the peak frequency of the gamma oscillations at DIV3 (Control Mdn 31.7 Hz, n = 47; 4AP Mdn 25.6 Hz, n = 15, p = 0.0054, Mann-Whitney U test). However, this drop in frequency was not accompanied by loss of PV phenotype, further substantiating that altered gamma oscillations and PV reduction are independent processes.

In contrast to the sustained effect of NMDAR inhibition on the frequency, in 4AP-exposed slices the gamma frequency recovered to control levels by DIV10 (Control Mdn 39.7 Hz, n = 38; 4AP Mdn 40.9 Hz, n = 20, p = 0.5392, Mann-Whitney U test) (Fig. 6c and e), along with an increase in the propensity to develop and maintain gamma oscillations. Responder rates doubled those observed in the control and APV treated groups (80%, 38% and 36% respectively). Interestingly, the increase in responder rates was similar to that displayed after prolonged oxidative stress due to BSO/Au exposure (DIV10) (70%, Fig. 6f). Thus, we propose that enhanced basal activity not only promoted interneuron survival, but in the long run also facilitated the induction of gamma oscillations.

## Discussion

Early life NMDAR hypofunction induces oxidative stress in interneurons, leading to decreased PV and GAD67 expression as well as disturbances in the fast spiking phenotype^1,25,45,46^. These changes are thought to underlie the aberrant gamma oscillations and the cognitive/behavioral symptoms associated with schizophrenia^13,14^. Although NMDAR hypofunction and thiol redox imbalance are common findings in this pathology, their individual effects on developing neural circuitries had not yet been examined.

**Figure 7:**
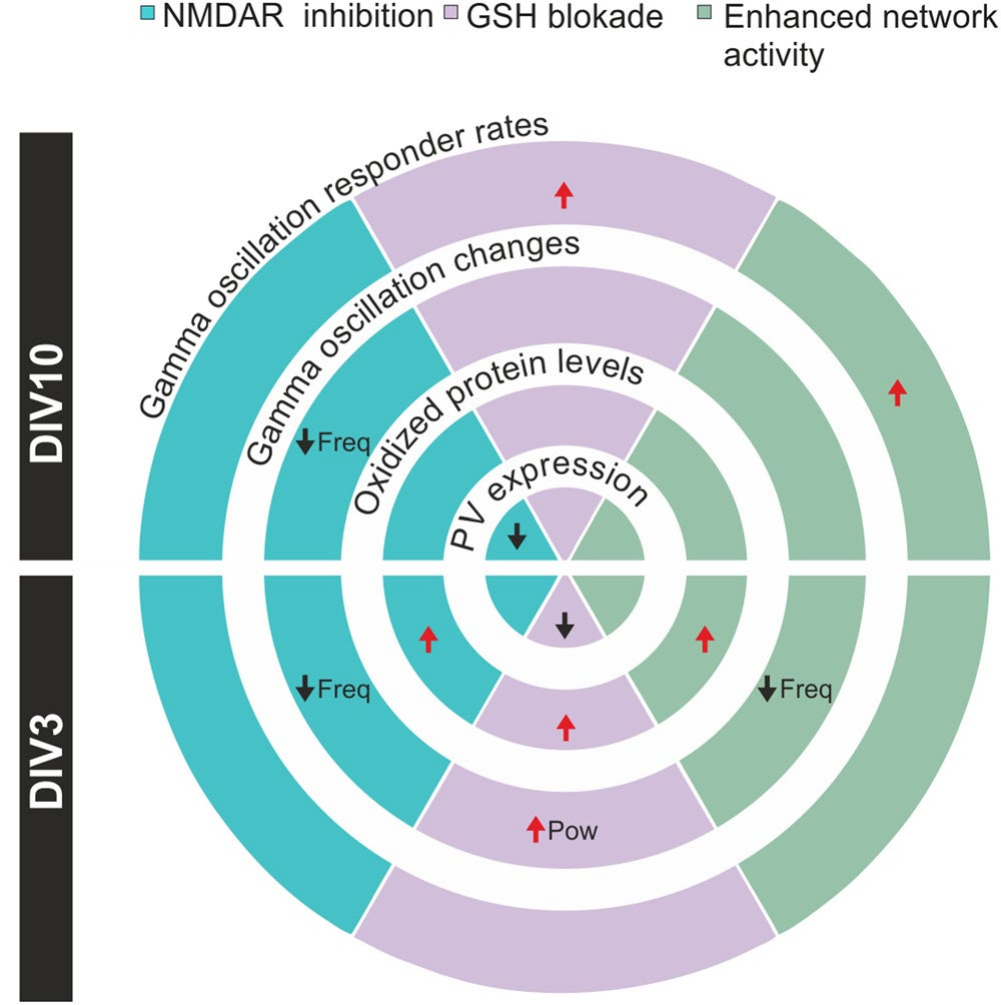
Summary of the changes in PV expression, protein oxidation, gamma oscillation properties and responder rates in the different treatment groups. Black arrows depict a significant decrease while red arrows indicate a significant increase. The segments without arrows were not statistically different from the control groups. The different treatments are depicted as follow: NMDAR-inhibition (APV exposure) in blue, oxidative stress (BSO/Au exposure) in purple and enhanced network activity (4AP exposure) in green.

Here we report that NMDAR inhibition and GSH-depletion during early postnatal development distinctively alter gamma oscillations. While both treatments resulted in oxidative stress and decreased PV expression, this could either coincide or follow the changes in the network activity. Pharmacologically enhanced network activity led to altered gamma oscillations and increased protein oxidation, yet PV expression remained unchanged. Together these findings indicate that PV expression and alterations of gamma-band activity are not mutually interdependent, and general oxidative stress cannot be the common denominator of this pathology (Fig. 7).

The use of hippocampal slice cultures in this study allowed for individual and graded manipulation of NMDAR activity or GSH levels during maturation of the interneurons, which takes place following the time point (P7) for explantation^28,32,47^. Indeed, the electrophysiological properties (AP duration, maximum frequency) of non-adapting interneurons and the gamma oscillation frequency and power stabilized after the first week *in vitro*. Moreover, the ratio of PV+ interneurons (Fig. 1c) as well as the properties of gamma band oscillatory activity corresponded those observed in age-matched acute slices, indicative of *ex vivo* interneuron and network maturation. Along with the progressive increase in power and frequency, the ability of slice cultures to maintain continuous gamma oscillations over 90 min declined with time *in vitro*. By DIV15, cultures displayed a higher propensity to shorter periods of oscillation intermingled with burst discharges. These changes could be due to the progressive neuron loss and increased axonal sprouting reported for aged slice cultures, which have been shown to facilitate the occurrence of epileptiform events^42,47,48^. Even though gamma oscillations are inducible in slice cultures up to DIV28^49^ we curtailed the study at DIV10 before this deviation from the acute slice preparation would appear. Bearing in mind the differences due to the cultivation, slice cultures still represent a useful tool to study network development under different stressors.

The tripartite combination of NMDAR hypofunction, decreased PV+ expression and altered gamma oscillations has been associated to schizophrenia pathology^1,25,45,46^. While the cell type unspecific NMDAR inhibition by ketamine or phencyclidine decreases PV and GAD67 expression^15,17,38^, selective genetic ablation of NMDAR on PV+ interneurons is sufficient to increase the power of gamma oscillations^16^ and to trigger schizophrenia-like behavior^50,51^.

Here we show that during early neuronal *in vitro* development, the exposure to a selective NMDAR inhibitior leads to an immediate and sustained decrease of the peak frequency of gamma oscillations, while reduction in PV expression occurs only after several days of treatment. This suggests that functional alterations in network activity might precede the downregulation of PV expression. The exact mechanisms by which NMDAR hypofunction disturb PV expression are not yet understood, however, oxidative stress has been suggested to contribute to PV loss^52^ either via the activation of NOX^13,53–55^ or by downregulating intrinsic antioxidant systems^51^. In our experimental setting, augmented levels of oxidized protein residues were observed following initial exposure to the NMDAR inhibitor, which, however, did not alter the PV expression. The loss in PV only became significant once the amount of oxidized protein had already recovered to control levels. This mismatch hints that general oxidative stress cannot be the main mechanism underlying the loss of PV phenotype.

GSH deficiency has frequently been reported in schizophrenia^1,19,21,22,53,56,57^ and might contribute to the altered network activity by redox modification of the NR1 and NR2A subunits leading to impaired NMDAR function^25,58–60^. However, in our model a partial (~45%) depletion of the GSH pool (in the presence of Trx inhibition) did not mimic the effect of NMDAR inhibition on the network activity, despite the immediate suppression of PV expression. In contrast to NMDAR inhibition, GSH depletion induced a temporary increase in power, while the frequency remained unchanged. In the long run (at DIV10), gamma oscillation power and frequency returned to the control values but the responder rate was significantly higher. This is in line with the observation that a similar (~30–40%) decrease of the brain GSH content following *in vivo* blockade of GSH synthesis results in impaired synaptic signaling and plasticity^60^ without having overt behavioral effects^61,62^. Thus decreased GSH content cannot account for the altered network activity observed in perinatal NMDAR hypofunction based models of schizophrenia. Remarkably, the increase in gamma power was only evident if GSH-depletion was applied during initial development of the cultures, whereas exposure to BSO after the first week *in vitro* (from DIV8 to 10) did not change gamma oscillation properties. The partial recovery of GSH levels from an initial ~45% reduction to a later ~30% in our model might come about from the upregulation of the antioxidant response element (ARE) genes inducing a compensatory GSH rise^63^. Another explanation might lay in the decrease of neuron/glia ratio (due to neuronal loss) increasing GSH availability as glial cells contain substantially more GSH than neurons^36^.

The conclusion that general oxidative stress cannot mechanistically explain the effects of NMDAR hypofunction is further supported by the fact that enhanced network activity via 4AP augmented oxidized protein levels and decreased the frequency, without altering PV expression. Interestingly, oxidative stress induced by GSH depletion or by 4AP was associated with an increased propensity to express stable gamma oscillations indicating alterations of the interneuron population. While NMDAR inhibition and GSH depletion, if anything, reduced the total number of interneurons, network activity enhancement with 4AP promoted their survival. Although, the ratio of PV+/YFP interneurons in long-term GSH depleted cultures recovered and even surpassed the control values, this rise was due to a decrease in the absolute numbers of interneurons rather than a genuine increase in the number of PV+ interneurons. The better survival rate of PV+ interneurons in face of GSH-depletion suggests that these cells are more resistant to oxidative stress, which might be ascribed to the protective effects of the particularly dense perineuronal nets (PNN) surrounding PV+ interneurons^64^. Despite the continuous presence of BSO, the truncated dendrites of PV+ cells observed at DIV3 recovered by DIV10 (Fig. 4a,b), accompanied by an increase in the gamma oscillation responder rates. While fully developed PNN are known to limit new synapse formation and reorganization, PNN ablation reinstates developmental plasticity^65,66^. Thus, PNN disintegration resulting from increased oxidative stress might explain the observed recovery of dendritic/axonal arborization and probably the enhanced propensity to gamma oscillations.

In case of enhanced network activity, the pro-survival effects of 4AP might arise from the increased synaptic NMDAR activation^25^, which might trigger neuroprotective effects such as increased BDNF expression^67^ and peroxide detoxification in neurons^24^. Thus, improved survival of interneurons in general or an increased ratio of PV+ interneurons will both result in higher responder rates.

There is a general agreement that NMDAR and redox dysfunction underlies the altered gamma oscillations in schizophrenia. In our hands, the initial and transient increase in the power of gamma oscillations together with the immediate decrease in PV expression elicited by GSH-depletion contrast with the sustained drop in gamma oscillation frequency and the late onset of PV loss observed after NMDAR inhibition. This suggests that NMDAR inhibition and GSH-depletion lead to divergent network and PV+ interneuron alterations, which might coexist but are not necessarily interdependent in the schizophrenia pathology.

## Methods

### Preparation of organotypic hippocampal slice cultures

Animal care and handling was in accordance with the Helsinki declaration and institutional guidelines (https://experimentelle-medizin.charite.de/). Slice culture preparation was proved by the State Office of Health and Social Affairs Berlin (https://www.berlin.de/lageso/), under the license number T0123/11. Data were obtained from ca. 750 brain slices from 150 animals. Each preparation included two animals and the slices were not differentiated between individuals. Slice cultures were prepared and maintained according to the Stoppini method^68^. Briefly, hippocampi from rats expressing venus-YFP on the VGAT promoter^30^ were extracted at postnatal day 6–7. Isolated hippocampi were aligned and cut into 400 µm slices perpendicular to the dorsoventral axis with a McIllvain Tissue Chopper. Slices were seeded on PTFE membranes (Millicell-CM, Millipore) and maintained in six-well plates filled with 1 mL culture medium (50% MEM, 25% HBSS, 25% Horse Serum and 1 mM L-glutamine, pH set to 7.3) in a humidified CO_2_ incubator (5% CO_2_) and left to recover for a day before any treatment.

### Pharmacology

Cultures were left to recover for a day after preparation. Drug application was started at DIV1 and was renewed with each change of medium (3 times per week) until the assessment time points (DIV3, 10 or 15). NMDAR inhibition was induced by DL-2-Amino-5-phosphonopentanoic acid (APV) (50 μM; Tocris), which has been shown to completely abolish NMDAR mediated responses in our preparation^69^. Induction of oxidative stress was achieved by co-application of the γ-glutamylcysteine synthetase inhibitor L-Buthionine sulfoximine (BSO, 0.5, 1, 5, 10 μM, Sigma-Aldrich), and by the thioredoxin reductase blocker Auranofin (Au, 1 μM; Sigma). Enhancement of network activity was induced with 4-Aminopyridine (4AP, 100 μM; Tocris), a non-selective blocker of voltage-activated K^+^ channels.

### Immunohistochemistry

Slice cultures were fixed with 4% paraformaldehyde/4% sucrose in phosphate-buffered saline (PBS) 0.1 M overnight at 4 °C and stored in 30% sucrose/PBS. For immunolabelling, slices were carefully detached from the PTFE membranes and processed free-floating. Incubation with a mouse derived anti-parvalbumin antibody (1:1000; Millipore) in 0.1% Triton X-100/PBS for 3 nights was followed by incubation over 24 hours with a goat anti-mouse Cy3 secondary antibody (1:100; Millipore). Slices were mounted on gold-coated slides and coverslipped with Vectashield HardSet Mounting Medium.

### Image acquisition

Fluorescence images were obtained with a spinning disk confocal microscope (Andor Revolution, BFIOptilas GmbH, Gröbenzell, Germany), equipped with an EMCCD camera (Andor iXonEM+), by using the 405, 491, and 561 nm laser lines for mBCI, YFP and Cy3, respectively. Z-stacks were obtained with 20× and 60× objectives (N.A. 0.5 and 1). Panorama images and cell quantification were conducted in 3D reconstructions created with ImageJ (Fiji release 1.51; Wayne Rasband, NIH, USA). Multicolor whole slice reconstructions were made with a NIKON A1R MP multiphoton microscope (25× N.A. 1.1 objective, Nikon, Amsterdam, The Netherlands) at the AMBIO Life Cell Imaging Core Facility (AMBIO.charite.de). Reconstructions of the hippocampal slices were made from tile scans (5 × 4 tiles, 10% overlap for the fitting algorhythm) covering the entire DG and cornu ammonis. The distance between focal planes was kept constant (1.2 µm) while the number of z stacks acquired varied depending on the thickness of the slices.

### GSH fluorescence quantification

Hippocampal slices were incubated for exactly 30 min with 50 µM monochlorobimane (mBCI), containing serum free medium, harvested from the PTFE membranes (9–18 slice cultures from 3–4 rats per treatment), followed by rapid freezing in liquid nitrogen. Cell lysates were obtained and the amount of protein was quantified by BSA assay and confirmed by Coomassie dye staining on a SDS-page gel loaded with 5 µg protein homogenate, as described elsewhere^70^. mBCI fluorescence – expressed as mean fluorescence units (MFUs) - was determined in a 96 well plate loaded with 50 µg protein homogenate in a Synergy HT microplate reader (Biotek) using 360 nm excitation and 528 nm emission over time (5–120 min). No difference of the outcome was observed over time.

### Oxidized protein quantification

Protein oxidation was measured by oxyblot assay (OxyBlot Protein Oxidation Detection Kit, Merck Chemicals), which labels the carbonyl groups introduced into proteins by oxidative reaction, by analyzing 7 µg protein homogenate and following the manufacture protocol. The densitometric analysis of the staining between 40 and 130 kDa was carried out with QuantityOne Software. Between 3 and 6 oxyblots were performed for each experiment, and their mean was used for further statistical analyses. To control the loading of equal amount of proteins in the representative oxyblot shown in Supplementary Fig. 2b, we performed a western blot assay on a 12% SDS-PAGE gel staining the ɑ4 subunit of proteasome, as described elsewhere^70^.

### Electrophysiology

The recording chambers were perfused with gassed (95% O_2_, 5% CO_2_, 1.3 ml per minute) artificial cerebrospinal fluid (aCSF) (in mM: NaCl 129, KCl 3, NaH_2_PO_4_ 1.25, MgSO_4_ 1.8, CaCl_2_ 1.6, NaHCO_3_ 26, glucose 10, pH 7.3). For submerged intracellular recordings, VGAT-YFP positive interneurons were identified using a NoranOz argon laser scanning confocal microscope (Prairie Technologies Inc. Middletown, WI) equipped with a 60× water-immersion objective. Glass micropipettes, filled with intracellular solution (in mM: K-gluconate 135, NaCl 4, CaCl_2_ 0,05, HEPES 10, EGTA 1, Mg-ATP 2) were used. Whole-cell patch clamp recordings were obtained using a Digidata 1320 digitizer, MultiClamp 700B amplifier and pCLAMP10 software, Molecular Devices (Sunnyvale, CA). Junction potential of −12 mV was not compensated for online. Access resistance (Ra), membrane resistance (Rm), membrane capacitance (Cm) and resting membrane potential (Em) were determined in voltage clamp (VC) mode. In current clamp (CC), pipette resistance was automatically compensated in the bridge balance mode. Consecutively increasing current steps of 25 pA and 1 s duration were applied and the evoked AP-trains evaluated. Discrimination of adapting and non-adapting interneurons were based on the interevent interval of the first and last 5 APs in a sequence. Individual AP properties were calculated from the second to last AP in a sequence upon a current step of 150 pA.

Local field potentials were recorded in Haas-type recording chamber using a EXB-EXT-02B NPI Electronic amplifier (Norbert Polder Instruments, Germany), high-pass filtered at 0.1 Hz, low-pass filtered at 1 kHz and sampled at 5 kHz by a digitizer CED Micro1401-2 with a ADC12 extension (Cambridge Electronic Design Limited, Cambridge, UK)^71^. Gamma oscillations were induced by adding Cch (5 µM) to the perfusion after 1000 s baseline and recorded from the CA3 pyramidal layer with glass pipettes filled with aCSF (<4 MΩ).

### Evaluation and statistics

Data processing of local field potential recordings was done using Spike2 (CED, Cambridge Electronic Design Limited, Cambridge, UK) and Matlab (The MathWorks Inc., 2013a). We computed the power spectral density of the Cch-induced gamma oscillations using Welch’s method on ten-second signal regions with a Hamming window with the length of 8192 samples. The gamma oscillation (20–80 Hz) median of the peak frequency and power was calculated from a 90 minute period (from 2000 to 7400 s) after the oscillation had stabilized. The comparison between groups (medians) was performed using the Kruskal-Wallis independent samples test (for more than two groups) and the Mann-Whitney U test (for two groups) in IBM SPSS Statistics 22 on the peak frequency and power in the 90 min (from 2000–7400 s) recording interval. Gamma oscillation power and frequency are presented in boxplots where the median and interquartile range are shown. For the representation of original traces, a low-pass second order Butterworth filter (500 Hz) was applied. The displayed power spectra were obtained from a 120 s interval where the 49–51 Hz frequency range was excluded. The analysis of the different activity patterns is presented as percentage, where the total number of recorded slices, including those displaying uninterrupted gamma oscillations, discontinuous gamma oscillations and no oscillations upon Cch application, are considered as 100%. Slice activity patterns were categorized as follows: gamma refers to slices displaying uninterrupted gamma oscillations in the 2000–7400 s (90 min) recording interval, partial gamma include those slices with discontinuous gamma oscillations during the same time interval and non-responder slices displayed basal activity but no gamma oscillations. The statistical analysis of the responder rates was performed using the two-tailed Fisher’s exact test taking into account the three categories of electrophysiological activity.

The quantification of the number of YFP+ (and PV+) interneurons was carried out in 3D reconstructions of the complete hippocampal slice using Arivis Vision 4D (arivis AG, Unterschleissheim, Germany) and its blobfinder analysis operator. For the images obtained with the confocal spinning disk microscope reported as part of Fig. 2c (P0, DIV15, P21/22), the stitching plugin of S. Preibisch *et al*.^72^ was used to create the hippocampal panorama pictures. On these reconstructions, PV+ interneurons were counted by hand using the Cell Counter plugin for Fiji (Madison, Wisconsin, USA) based on 64 bit ImageJ distribution (Wayne Rasband, NIH), while VGAT-YFP interneurons were counted using the Analyze Particles plugin.

As the thickness of the cultures varied from slice to slice, we decided to normalize the total number of YPF+ and PV+ interneurons by dividing them with the number of focal planes necessary to scan the entire z-depth from top to bottom (all regions of the hippocampus included). In the figures, the median of the YFP+ and PV+ interneurons per focal plane are presented. However, the ratio of PV+/YFP+ interneurons within a given slice culture was calculated considering the entire slice (all focal planes) and the total YFP+ and PV+ interneuron count. For each treatment and developmental time-point 10 to 20 hippocampal slices obtained from 3–10 rats were analyzed. Size variations in X-Y directions between individual cultures were kept at a minimum due to the alignment of the hippocampi during the preparation. Equal distribution of PV+ cells along the dorsoventral axis of the hippocampus was verified in cultures sliced parallel to the length axis. Statistical evaluation was performed using the Mann-Whitney U test in IBM SPSS version 22.

To assess the GSH and oxidized protein levels, the mean and SD of different experiments were obtained merging slices derived from different rats (n = 3–4 for each experiment and treatment). The significant differences between groups are reported as p values, and were computed by One-way Anova test by applying the Bonferroni correction method for multiple comparisons. Data were tested for normality distribution and homoscedasticity by Shapiro-Wilk and Levene tests, respectively.

### Data availability

The datasets generated and analyzed during the current study are available from the corresponding author on reasonable request.

## Electronic supplementary material

Supplementary material
