# Author Correction: NMDA-receptor inhibition and oxidative stress during hippocampal maturation differentially alter parvalbumin expression and gamma-band activity

**DOI:** 10.1038/s41598-018-31011-6

**Published:** 2018-08-29

**Authors:** Luisa A. Hasam-Henderson, Grace C. Gotti, Michele Mishto, Constantin Klisch, Zoltan Gerevich, Jörg R. P. Geiger, Richard Kovács

**Affiliations:** 1 Institut für Neurophysiologie, Charité – Universitätsmedizin Berlin, corporate member of Freie Universität Berlin, Humboldt-Universität zu Berlin, and Berlin Institute of Health, Berlin, Charité Platz 1, 10117 Berlin, Germany; 2 The NeuroCure Cluster of Excellence, Berlin, Germany; 3 Institut für Biochemie, Charité – Universitätsmedizin Berlin, corporate member of Freie Universität Berlin, Humboldt-Universität zu Berlin, and Berlin Institute of Health, Berlin, Charité Platz 1, 10117 Berlin, Germany; 4 Centre for Inflammation Biology and Cancer Immunology (CIBCI) & Peter Gorer Department of Immunobiology, King’s College London, SE1 1UL London, United Kingdom

Correction to: *Scientific Reports* 10.1038/s41598-018-27830-2, published online 22 June 2018

The Acknowledgements section in this Article is incomplete.

“The study was supported by the DFG grant Ko3814/1-1 to L.A.H.-H., G.C.G. and R.K.”

should read:

“The study was supported by the DFG grant Ko3814/1-1 to L.A.H.-H., G.C.G. and R.K. The authors sincerely thank Christin Keller for her assistance with the oxyblot recordings and Peter Kloetzel for the Financial Support of M.M. through the Berlin Institute of Health (BIH) Project CRG1 TP1 12.01.111.”

