# Supplementary material for: NMDA-receptor inhibition and oxidative stress during hippocampal maturation differentially alter parvalbumin expression and gamma-band activity
Source: Sci Rep. 2018 Jun 22;8:9545. doi: 10.1038/s41598-018-27830-2 (PMC6015018; doi:10.1038/s41598-018-27830-2)
Supplement: Supplementary file 1 — Supplementary material [file 41598_2018_27830_MOESM1_ESM.pdf]

Supplementary material for the manuscript "*NMDA-receptor inhibition and oxidative stress during hippocampal maturation differentially alter parvalbumin expression and gamma-band activity*"

Autors: Luisa A. Hasam-Henderson[1], Grace C. Gotti[1], Michele Mishto[3,4] Constantin Klisch[1], Zoltan Gerevich[1], Jörg R.P. Geiger[1,2], Richard Kovács[1]*

Correspondence to richard.kovacs@charite.de

Supplementary I. Maturation of the electrophysiological properties of adapting and non-adapting interneurons

Boxplots representing the changes in the electrophysiological properties of adapting and non-adapting YFP-VGAT interneurons depending on the respective age in culture (DIV 2-5, 6-8, 9-11, 12-15, 16-17): **a.** Membrane resistance, **b.** Membrane capacitance, **c.** Frequency of the action potentials upon a 1 s depolarizing step and **d.** Action potential half-width. **e.** The number of recorded cells (165 cells) for both interneuron groups are listed on the right table. These cells were recorded from 70 slices, prepared from 32 animals.

Supplementary II. Neuronal and glial monochlorobimane fluorescence pattern and time-dependent changes in oxidized protein levels in organotypic hippocampal slice cultures

Representative confocal images of the CA3 region of DIV10 (P7) slice cultures stained with monochlorobimane (mBCL) (10 µM) in the perfusion, depicting VGAT-YFP interneurons (green) and mBCL (magenta) labelled cells. Note that mBCL fluorescence pattern resembles the distribution of glial cells in slice cultures whereas VGAT-YFP cells were only weakly stained by mBCL. Image stacks were taken with a 20x (upper row, scale bar corresponds to 100 µm) or 60x objective (lower row, scale bar corresponds to 20 µm). **b.** Representative oxyblot (of 2 independent experiments) of cell lysates of DIV0, 4, 7 14 slice cultures. To control the loading of equal amount of proteins, we performed in parallel a western blot assay on a 12 % SDS-PAGE gel staining the α4 subunit of proteasome.

Supplementary III. Full length gels corresponding to the figure 3 titled "Effects of NMDAR inhibition, oxidative stress and increased synaptic activity on total GSH content and on protein oxidation levels"

Representative oxyblot (of 3-4 independent experiments) presenting oxidized protein level in cell lysates at DIV3 (left) and 10 (right) in control and treated slice cultures. Different time exposures were not applied in the presented data. In the figure 3 of the main text, the last gel line was not shown since this treatment was not further addressed in the manuscript.

Supplementary Figure I

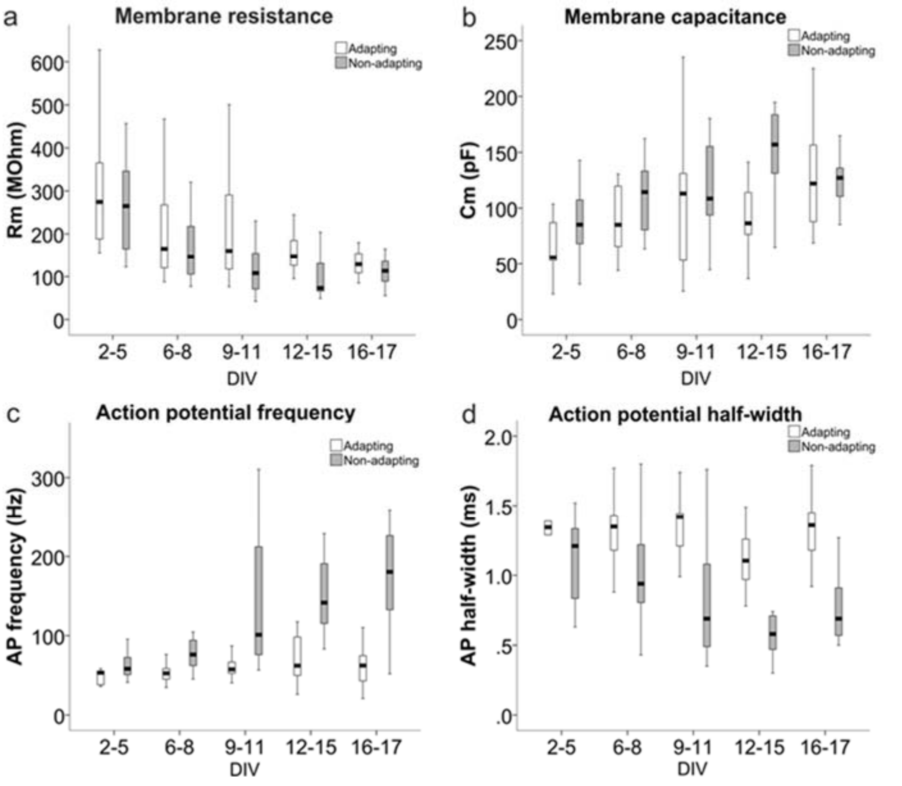

Number of recorded cells per developmental time point

Days in vitro	Adapting (n)	Non-adapting (n)
2-5	7	16
6-8	26	16
9-11	15	21
12-15	16	21
16-17	13	14

Supplementary Figure II

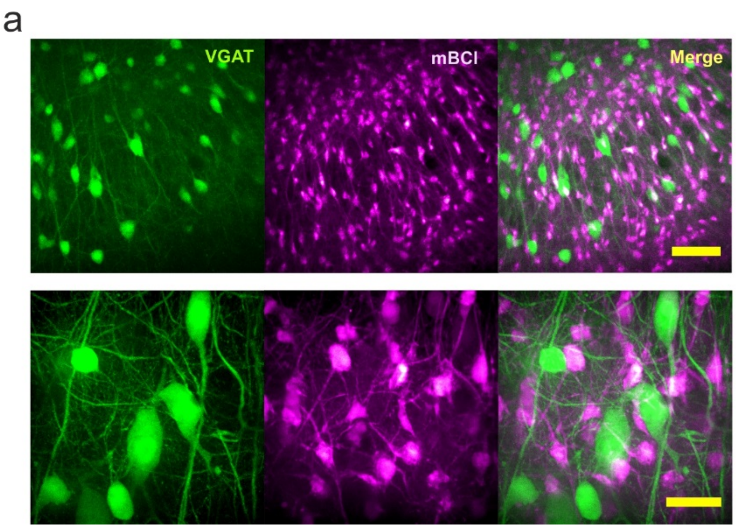

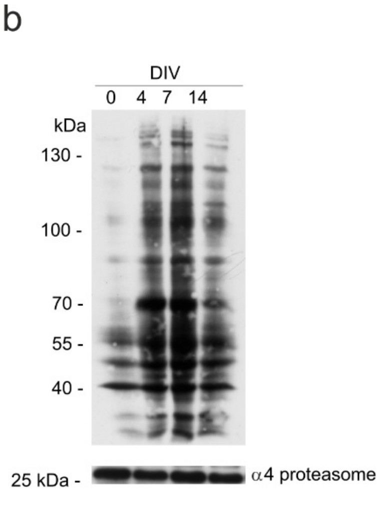

Supplementary Figure III

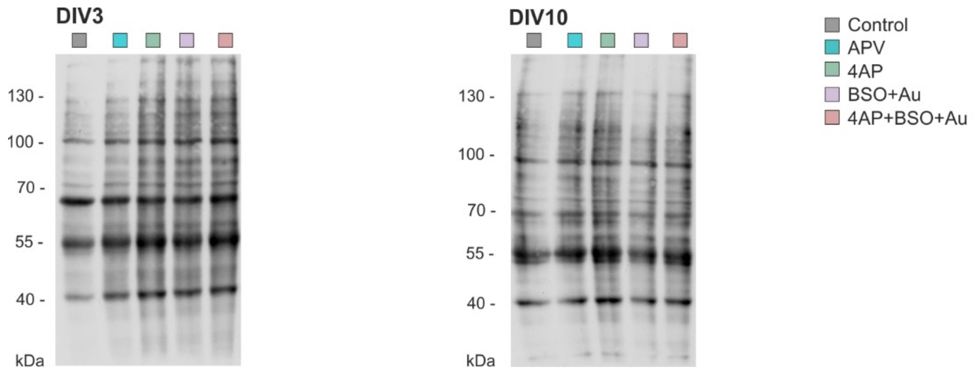

Descriptive statistics and statistical tests

Figure 2.

Figure 2d. Gamma oscillation frequency

Slices (n)	DIV	Median (Hz)	Mean (Hz)	SEM (Hz)
47	DIV3	31,73	31,3682	0,54408
38	DIV10	39,67285156	40,00212017	0,972733621
10	DIV15	41,35131836	40,98510742	1,20195186

Kruskal Wallis Test $p = 4{,}6703^{-11}$ $H(2) = 47.574$ df=2

Mann Whitney U (2 tailed)

Compared groups	p	U	Z	N	r
**DIV3 vs DIV10**	$p = 5{,}6252^{-10}$	192,0	-6,201	85	0,457705998
**DIV3 vs DIV15**	$p = 4{,}2113^{-6}$	16,0	-4,601	57	0,377967822
**DIV10 vs DIV15**	p= 0,454	160,5	-,750	48	0,01195241

Figure 2e. Gamma oscillation power

Slices (n)	DIV	Median (μV²)	Mean (μV²)	SEM (μV²)
47	DIV3	1,33635	3,217540915	0,822984922
38	DIV10	4,923105	51,40300095	22,19897095
10	DIV15	3,059635	8,6367301	3,859603216

Kruskal Wallis Test p= 0,007 $H(2) = 9.936$ df=2

Mann Whitney U (2 tailed)

Compared groups	p	U	Z	N	r
**DIV3 vs DIV10**	$p = 1{,}5445^{-5}$	404,0	-4,322	85	0,222402555
**DIV3 vs DIV15**	p=0,036	135,0	-2,098	57	0,078608112
**DIV10 vs DIV15**	p=0,402	157,0	-,838	48	0,014932468

Figure 3.

Figure 3b left

Descriptives

	N	Mean	Std. Dev	Std. Error
**Control**	6	100	0	0
**APV**	5	102,8	8,66412	3,87471
**4AP**	4	96,55	12,00014	6,00007
**BSO/Au**	4	55,3	4,32281	2,1614

Tests of Normality

	Kolmogorov-Smirnova			Shapiro-Wilk		
	Statistic	df	Sig.	Statistic	df	Sig.
**APV**	0,322	5	0,098	0,85	5	0,194
**4AP**	0,282	4	.	0,939	4	0,65
**BSO/Au**	0,234	4	.	0,94	4	0,653

ANOVA $F(3,15)=40.049$, $p=2.12^{-7}$

Bonferroni		Mean Difference	Std. Error	Sig.	95% Confidence Interval	
					Lower Bound	Upper Bound
**Control vs**	**APV**	-2,08000	4,16746	1,000	-15,4016	11,2416
	**4AP**	3,45000	4,44253	1,000	-10,7509	17,6509
	**BSO/Au**	44,7*	4,44253	,000	30,4991	58,9009

*. The mean difference is significant at the 0.05 level.

Figure 3b right

Descriptives

	N	Mean	Std. Deviation	Std. Error
**Control**	7	100	0	0
**APV**	5	93	14,43295	6,45461
**4AP**	4	95,425	16,18773	8,09386
**BSO/Au**	5	72,28	8,89028	3,97585

Tests of Normality

	Kolmogorov-Smirnova			Shapiro-Wilk		
	Statistic	df	Sig.	Statistic	df	Sig.
**APV**	0,248	5	0,2	0,887	5	0,34
**4AP**	0,385	4	.	0,729	4	0,024
**BSO/Au**	0,236	5	0,2	0,902	5	0,419

ANOVA $F(3, 17)=7.077$, $p=0.002714$

Bonferroni		Mean Difference	Std. Error	Sig.	95% Confidence Interval	
					Lower Bound	Upper Bound
**Control vs**	**APV**	7	5,98797	1	-11,7735	25,7735
	**4AP**	4,575	6,40974	1	-15,5209	24,6709
	**BSO/Au**	27,72000*	5,98797	0,001	8,9465	46,4935

*. The mean difference is significant at the 0.05 level.

Figure 3d left

Descriptives

	N	Mean	Std. Deviation	Std. Error
**Control**	5	100	0	0
**APV**	4	126,825	11,52139	5,7607
**4AP**	4	144,1125	19,1449	9,57245
**BSO/Au**	3	141,2	3,95095	2,28108

Tests of Normality

	Kolmogorov-Smirnova			Shapiro-Wilk		
	Statistic	df	Sig.	Statistic	df	Sig.
**APV**	0,258	4	.	0,948	4	0,704
**4AP**	0,271	4	.	0,83	4	0,168
**BSO/Au**	0,324	3	.	0,877	3	0,316

ANOVA F(3,12)=17.086, p=0.000309

Bonferroni		Mean Difference	Std. Error	Sig.	95% Confidence Interval	
					Lower Bound	Upper Bound
**Controls vs**	**APV**	-26,82500*	7,41396	0,028	-51,4816	-2,1684
	**4AP**	-44,11250*	7,41396	0,0000355	-68,7691	-19,4559
	**BSO/Au**	-41,20000*	8,07129	0,002	-68,0427	-14,3573

*. The mean difference is significant at the 0.05 level.

Figure 3d right

Descriptives

	N	Mean	Std. Deviation	Std. Error
Control	5	100	0	0
APV	5	112,1	26,33458	11,77718
4AP	3	149,7333	40,20526	23,21252
BSO/Au	3	126,5667	23,18972	13,38859

Tests of Normality

	Kolmogorov-Smirnova			Shapiro-Wilk		
	Statistic	df	Sig.	Statistic	df	Sig.
APV	0,222	5	,200*	0,887	5	0,343
4AP	0,191	3	.	0,997	3	0,9
BSO/Au	0,194	3	.	0,997	3	0,888

*. This is a lower bound of the true significance.

ANOVA F(3,12)=2.844, p=0.082389

Bonferroni		Mean Difference	Std. Error	Sig.	95% Confidence Interval	
					Lower Bound	Upper Bound
**Control vs**	**APV**	-12,1	14,36134	1	-59,8614	35,6614
	**4AP**	-49,73333	16,58304	0,096	-104,8835	5,4168
	**BSO/Au**	-26,56667	16,58304	1	-81,7168	28,5835

Figure 4

Figure 4b left

Number of YFP+ Interneurons per image stack							Mann Whitney U 2 tailed on the medians				
		Slices (n)	Mean	SEM	Median		p	U	Z	N	r
DIV3	Control	15	122,8687	9,63425	111,7500		,659	74,0	-,441	26	0,0077845
	APV	11	128,8544	11,58166	113,1818						
DIV10	Control	13	76,6039	6,23326	80,5000		,063	35,0	-1,861	23	0,1573427
	APV	10	62,3585	4,13014	61,8042						

Figure 4b middle

Number of PV+ Interneurons per image stack							Mann Whitney U 2 tailed on the medians				
		Slices (n)	Mean	SEM	Median		p	U	Z	N	r
DIV3	Control	15	5,4734	,40623	5,3438		,177	56,5	-1,350	26	0,0728599
	APV	11	4,3319	,66509	4,5357						
DIV10	Control	13	13,3009	1,18541	11,7500		,009	23,0	-2,605	23	0,3083916
	APV	10	8,5952	,64085	8,9465						

Figure 4b right

Ratio of YFP+/PV+ interneurons in 3D slice reconstructions							Mann Whitney U 2 tailed on the medians				
		Slices (n)	Mean	SEM	Median		p	U	Z	N	r
DIV3	Control	15	4,7572	0,4874967	4,318363		,243	60,0	-1,168	26	0,0545455
	APV	11	3,4918	,47414	4,0562						
DIV10	Control	13	17,647925	0,9011611	16,766039		,026	29,0	-2,233	23	0,2265734
	APV	10	14,0601	1,14856	13,0744						

Figure 4c. DIV3 Gamma oscillations

		Frequency			Power		
Slices (n)	DIV	Median (Hz)	Mean (Hz)	SEM (Hz)	Median (μV^2)	Mean (μV^2)	SEM (μV^2)
47	DIV3 Control	31,73	31,3682	0,54408	1,33635	3,2175409	0,82298492
14	DIV3 APV	27,1606445	27,85818	1,044708	1,7648	6,0086807	2,26815236

Mann Whitney U (2 tailed)

		p	U	Z	N	r
DIV3 Control vs APV	Frequency	,003	157,0	-2,955	61	0,457706
	Power	,320	271,0	-,995	61	0,0164918

Figure 4e. DIV10 Gamma oscillations

		Frequency			Power		
**Slices (n)**	**DIV**	**Median (Hz)**	**Mean (Hz)**	**SEM (Hz)**	**Median (μV^2)**	**Mean (μV^2)**	**SEM (μV^2)**
38	DIV10 Control	39,67285156	40,00212	0,9727336	4,923105	51,4030009	22,19897095
20	DIV10 APV	36,62109376	35,964966	1,2327454	0,138525	113,592076	0,56004521

Mann Whitney U (2 tailed)

		p	U	Z	N	r
**DIV10 Control vs APV**	**Frequency**	,025	243,5	-2,235	58	0,08763553
	**Power**	,326	320,0	-,982	58	0,0169022

Figure 5.

Figure 5b left

Number of YFP+ Interneurons per image stack							Mann Whitney U 2 tailed on the medians				
		Slices (n)	Mean	SEM	Median		p	U	Z	N	r
DIV3	Control	15	122,8687	9,63425	111,7500		,407	73,0	-,830	27	0,0264652
	BSO/Au	12	134,6937	12,43498	128,1353						
DIV10	Control	13	76,6039	6,23326	80,5000		,277	58,0	-1,088	25	0,04930966
	BSO/Au	12	69,2854	9,83680	59,0977						

Figure 5b middle

Number of PV+ Interneurons per image stack							Mann Whitney U 2 tailed on the medians				
		Slices (n)	Mean	SEM	Median		p	U	Z	N	r
DIV3	Control	15	5,4734	,40623	5,3438		,011	38,0	-2,537	27	0,24761905
	BSO/Au	12	3,8426	,40684	4,2294						
DIV10	Control	13	13,3009	1,18541	11,7500		,786	73,0	-0,272	25	0,00308185
	BSO/Au	12	12,8219	1,19028	11,9344						

Figure 5b right

Ratio of YFP+/PV+ interneurons in 3D slice reconstructions							Mann Whitney U 2 tailed on the medians				
		Slices (n)	Mean	SEM	Median		p	U	Z	N	r
DIV3	Control	15	4,7572	0,48749669	4,31836301		,010	37,0	-2,586	27	0,00308185
	BSO/Au	12	3,0218	,36910	2,9805						
DIV10	Control	13	17,647925	0,90116107	16,7660386		,019	31,0	-2,346	25	0,22940559
	BSO/Au	12	21,6397	1,14088	21,6517						

Figure 5c. DIV3 Gamma oscillations

		Frequency			Power		
Slices (n)	DIV	Median (Hz)	Mean (Hz)	SEM (Hz)	Median (μV^2)	Mean (μV^2)	SEM (μV^2)
47	DIV3 Control	31,73	31,3682	0,54408	1,33635	3,2175409	0,8229849
35	DIV3 BSO/Au	30,517578	30,142648	0,575245	2,39368	9,1434389	2,3914226

Mann Whitney U (2 tailed)

		p	U	Z	N	r
DIV3 Control vs BSO/Au	Frequency	,168	675,5	-1,380	82	0,0235189
	Power	,027	587,0	-2,208	82	0,0601775

Figure 5e. DIV10 Gamma oscillations

		Frequency			Power		
**Slices (n)**	**DIV**	**Median (Hz)**	**Mean (Hz)**	**SEM (Hz)**	**Median (μV^2)**	**Mean (μV^2)**	**SEM (μV^2)**
38	DIV10 Control	39,672852	40,00212	0,9727336	4,923105	51,403001	22,198971
19	DIV10 BSO/Au	40,588379	40,475946	1,6038755	7,90862	107,24702	64,250692

Mann Whitney U (2 tailed)

		p	U	Z	N	r
**DIV10 Control vs BSO/Au**	**Frequency**	,912	354,5	-0,110	57	0,0002165
	**Power**	0,747730	342	-0,32163	57	0,2224026

Figure 5g. DIV10 Gamma oscillations

		Frequency			Power		
**Slices (n)**	**DIV**	**Median (Hz)**	**Mean (Hz)**	**SEM (Hz)**	**Median (μV^2)**	**Mean (μV^2)**	**SEM (μV^2)**
38	DIV10 Control	39,672852	40,00212	0,9727336	4,923105	51,403001	22,198971
13	DIV8-10 BSO/Au	44,555664	42,583759	1,8155396	5,11695	15,192163	5,0363188

Mann Whitney U (2 tailed)

		p	U	Z	N	r
**DIV10 Control vs DIV8-10 BSO/Au**	**Frequency**	,122	175,5	-1,547	51	0,0478642
	**Power**	0,4891681	215	-0,69163	51	0,0095671

Figure 6.

Figure 6b left

Number of YFP+ Interneurons per image stack							Mann Whitney U 2 tailed on the medians				
		Slices (n)	Mean	SEM	Median		p	U	Z	N	r
DIV3	Control	15	122,8687	9,63425	111,7500		,023	34,0	-2,274	25	0,21551282
	4AP	10	160,2690	13,62696	163,7538						
DIV10	Control	13	76,6039	6,23326	80,5000		,005	23,0	-2,810	24	0,34328975
	4AP	11	109,3089	8,27164097	111,3846						

Figure 6b middle

Number of PV+ Interneurons per image stack							Mann Whitney U 2 tailed on the medians				
		Slices (n)	Mean	SEM	Median		p	U	Z	N	r
DIV3	Control	15	5,4734	,40623	5,3438		1,000	75,0	0,000	25	0
	4AP	10	5,6583	,54493	5,3287						
DIV10	Control	13	13,3009	1,18541	11,7500		,060	39,0	- 1,883	24	0,15416039
	4AP	11	16,7141	1,02088	16,7805						

Figure 6b right

Ratio of YFP+/PV+ interneurons in 3D slice reconstructions							Mann Whitney U 2 tailed on the medians				
		Slices (n)	Mean	SEM	Median		p	U	Z	N	r
DIV3	Control	15	4,7572	0,48749669	4,31836301		,108	46,0	-1,609	25	0,10782051
	4AP	10	3,7878	,53975	3,2383						
DIV10	Control	13	17,647925	0,90116107	16,7660386		,369	56,0	-0,898	24	0,03506233
	4AP	11	16,1777	1,42967	14,8205						

Figure 6c. DIV3 Gamma oscillations

		Frequency			Power		
Slices (n)	DIV	Median (Hz)	Mean (Hz)	SEM (Hz)	Median (μV^2)	Mean (μV^2)	SEM (μV^2)
47	DIV3 Control	31,73	31,3682	0,54408	1,33635	3,2175409	0,8229849
15	DIV3 4AP	25,634766	27,567546	1,0949397	1,45013	4,2516489	1,8913713

Mann Whitney U (2 tailed)

		p	U	Z	N	r
**DIV3 Control vs 4AP**	**Frequency**	,005	183,5	-2,783	62	0,1269649
	**Power**	0,675109	327	-0,41914	62	0,0028801

Figure 6e. DIV10 Gamma oscillations

		Frequency			Power		
**Slices (n)**	**DIV**	**Median (Hz)**	**Mean (Hz)**	**SEM (Hz)**	**Median (μV^2)**	**Mean (μV^2)**	**SEM (μV^2)**
38	DIV10 Control	39,672852	40,00212	0,9727336	4,923105	51,403001	22,198971
20	DIV10 4AP	40,893555	41,33606	1,4798045	13,54435	45,314111	25,237672

Mann Whitney U (2 tailed)

		p	U	Z	N	r
**DIV10 Control vs 4AP**	**Frequency**	,539	342,5	-0,614	58	0,0066128
	**Power**	0,3596112	324	-0,9161	58	0,0147237
